# Supplementary material for: Killing by Type VI secretion drives genetic phase separation and correlates with increased cooperation
Source: Nat Commun. 2017 Feb 6;8:14371. doi: 10.1038/ncomms14371 (PMC5303878; doi:10.1038/ncomms14371)
Supplement: Supplementary Information — Supplementary Figures, Supplementary Tables and Supplementary References [file ncomms14371-s1.pdf]

Supplementary Figures

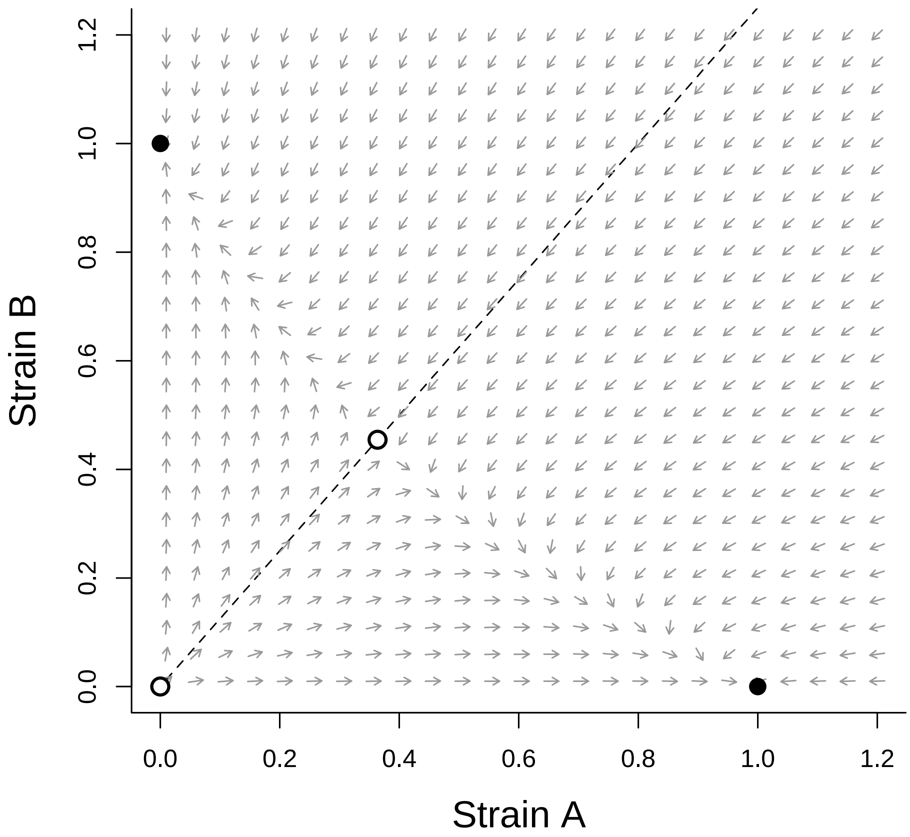

Supplementary Figure 1. Illustration of T6SS mediated dynamics in a well-mixed population. Arrows indicate the direction of the dynamics for any given combination of densities of strains *A* and *B*. Open circles indicate the unstable equilibria, while closed circles are stable equilibria. The dashed line indicates the critical ratio of strains *A* and *B* dictating which stable equilibrium will be reached. Parameter values are r = 2, s = 2, α_{AB} = 0.8 and α_{BA} = 1. Note that for these parameter the basin of attraction for the equilibrium where *A* dominates ($A = r/s$, B = 0) is larger than the basin of attraction for the equilibrium where *B* dominates (A = 0, $B = r/s$) owing to *A*'s superior killing ability ($\alpha_{BA} > \alpha_{AB}$).

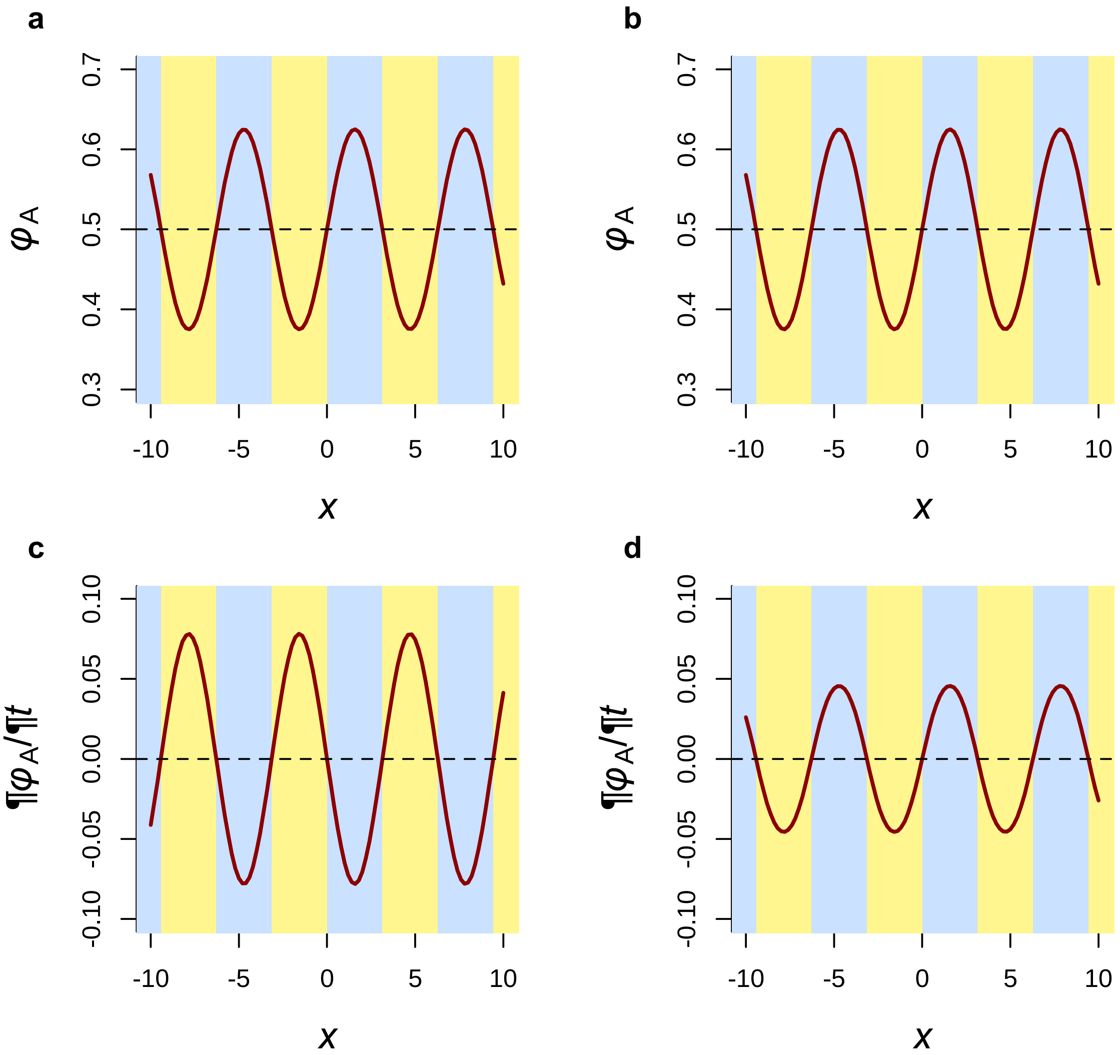

Supplementary Figure 2. Illustration of spatial decomposition of mixed equilibrium in one dimension. Plotted are the proportion of cells of strain A (φ_A) in (a) and (b), and the rate of change in this proportion ($\partial\varphi_A/\partial t$) in (c) and (d) along a spatial axis *x*. The different shaded regions indicate regions of positive (blue) and negative (yellow) fluctuation in composition. In (a) and (c) a high rate of bacterial dispersal/diffusion (d = 1) through space means that the fluctuation in the density of A is on narrow a scale relative to bacterial dispersal/diffusion and the system will return towards the spatially homogenous equilibrium ($\partial\varphi_A/\partial t$ and φ_A are of opposite sign) as bacterial/dispersal diffusion is strongly homogenising. However, in (b) and (d) when the bacterial dispersal/diffusion rate is lower (d = 0.01) the spatial fluctuations occur on a wider scale relative to bacterial dispersal/diffusion and the system will be begin to decompose into areas divergent in composition ($\partial\varphi_A/\partial t$ and φ_A are of opposite sign) as bacterial dispersal/diffusion is a relatively weak force compared to the demographic effects of killing. Other parameter values are r = 2, s = 2, α_{AB} = 1, α_{BA} = 1, a = 0.1 and β = 1.

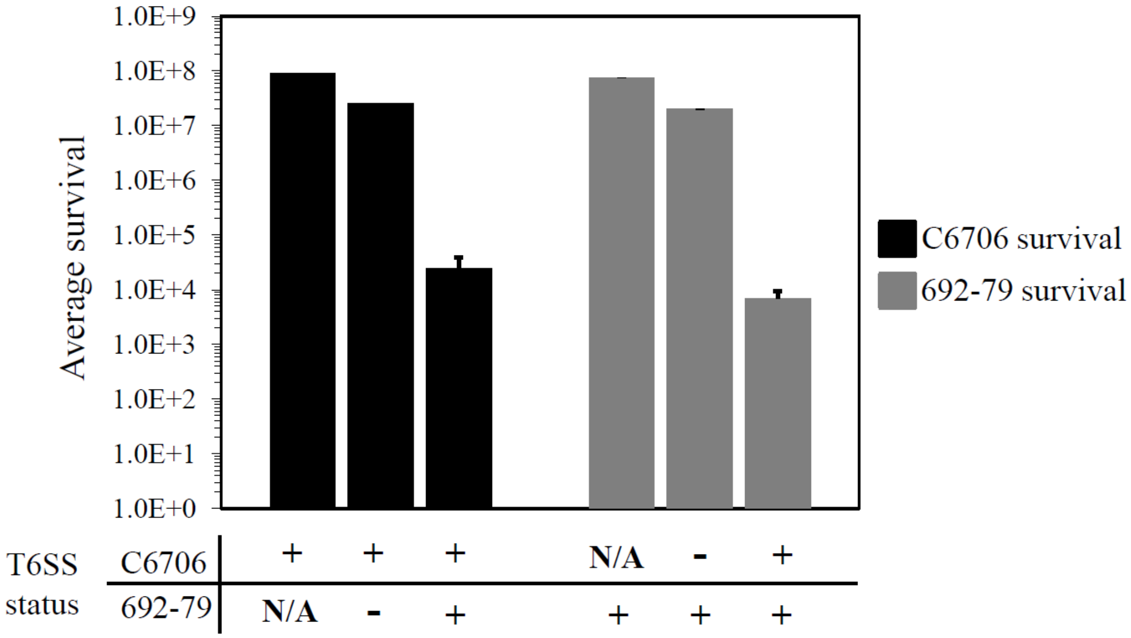

Supplementary Figure 3. *Vibrio cholerae* strains C6706 and 692-79 are mutual killers. Spectinomycin resistant C6706 survival was measured after a 3 hour incubation on membrane filters on LB agar in a 1:10 ratio with LB broth[1], or liquid cultures of 692-79 $T6SS^-$ (Δ*vasK*), or 692-79 $T6SS^+$ strains and is represented by black bars. Kanamycin resistant 692-79 survival was measured similarly in a 1:10 ratio with LB broth, C6706 $T6SS^-$ (Δ*vask*), or C6706 $T6SS^+$ and is represented by gray bars. Shown are average prey survival values ± one standard deviation after triplicate encounters. One representative experiment is shown of three performed.

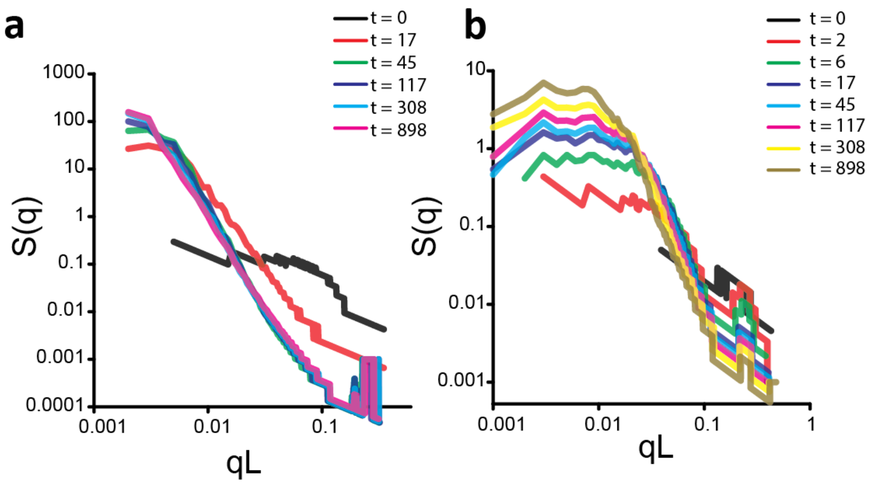

Supplementary Figure 4. Structural analysis of additional models. The static structure factor, *S(q)*, is plotted at different times versus wavenumber, *q*, multiplied by cell size, *L*, for the PDE model with diffusion *d* = 0.01 (a) and the Ising model with Glauber spin flip dynamics on the square lattice (b). In both the PDE and Ising spin models, *S(q)* increases at small *q* as time increases, just as was seen for the individual based model and experiments in Figure 2a and b, respectively**.**

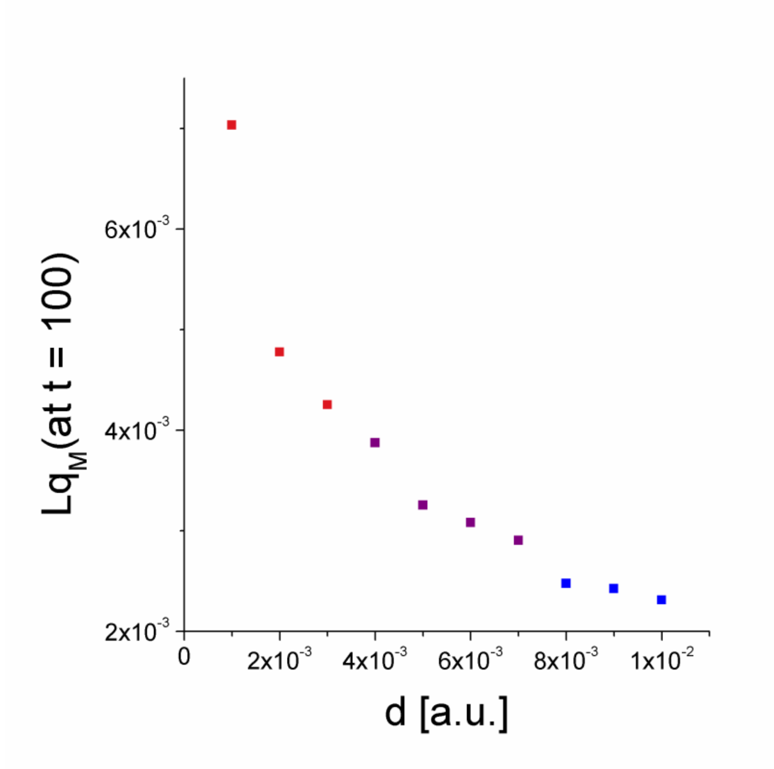

Supplementary Figure 5. Cellular mobility speeds decomposition in the PDE model. For the PDE model, the value of q_M after 100 time steps, multiplied by cell size, *L*, is plotted vs. the rate of cellular mobility (random movement akin to diffusion), *d*. As *d* increases, the value of q_M decreases, indicating that the demixing process is further along.

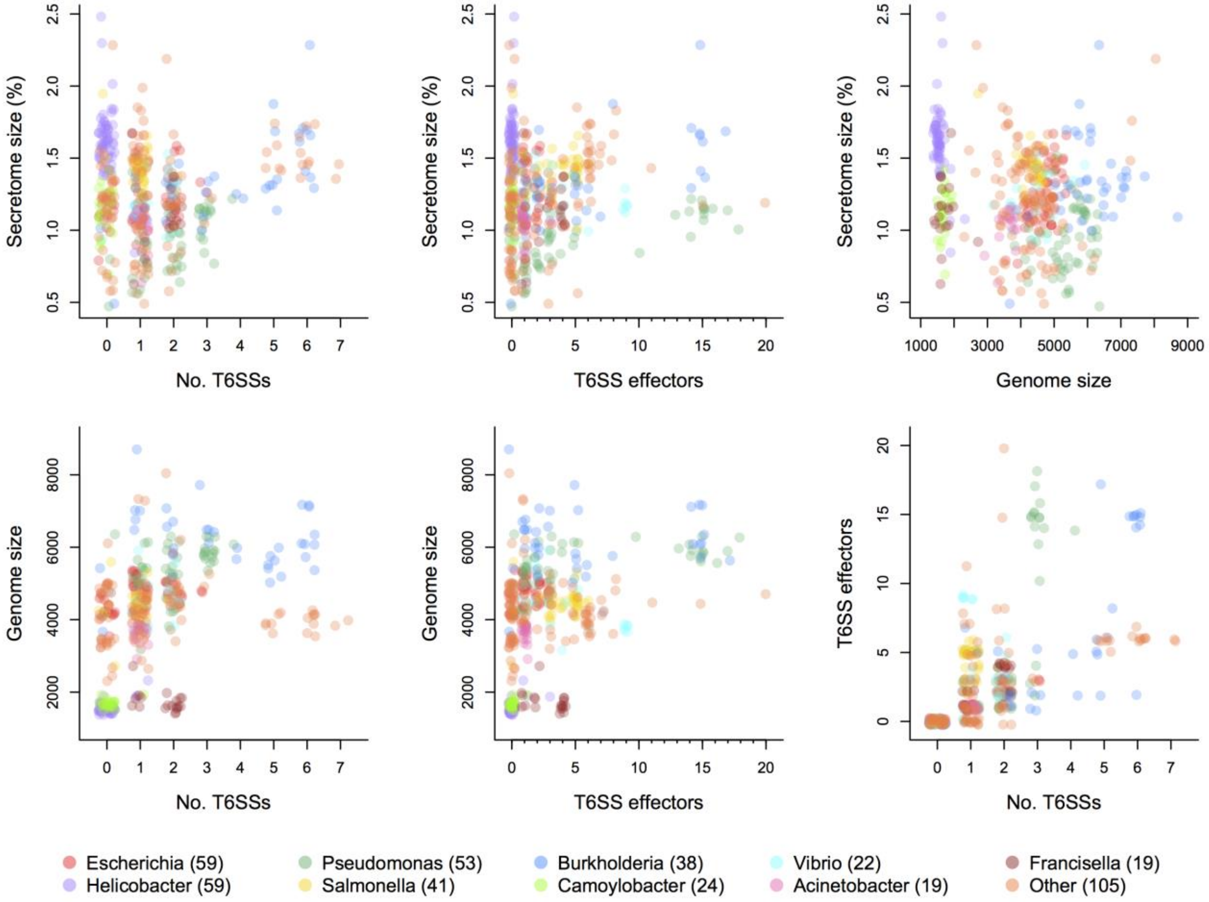

Supplementary Figure 6. Scatterplots of raw data from comparative analysis. Shown are pairwise scatterplots of all data included in our comparative analysis. Colours indicate different genera as indicated in the legend.

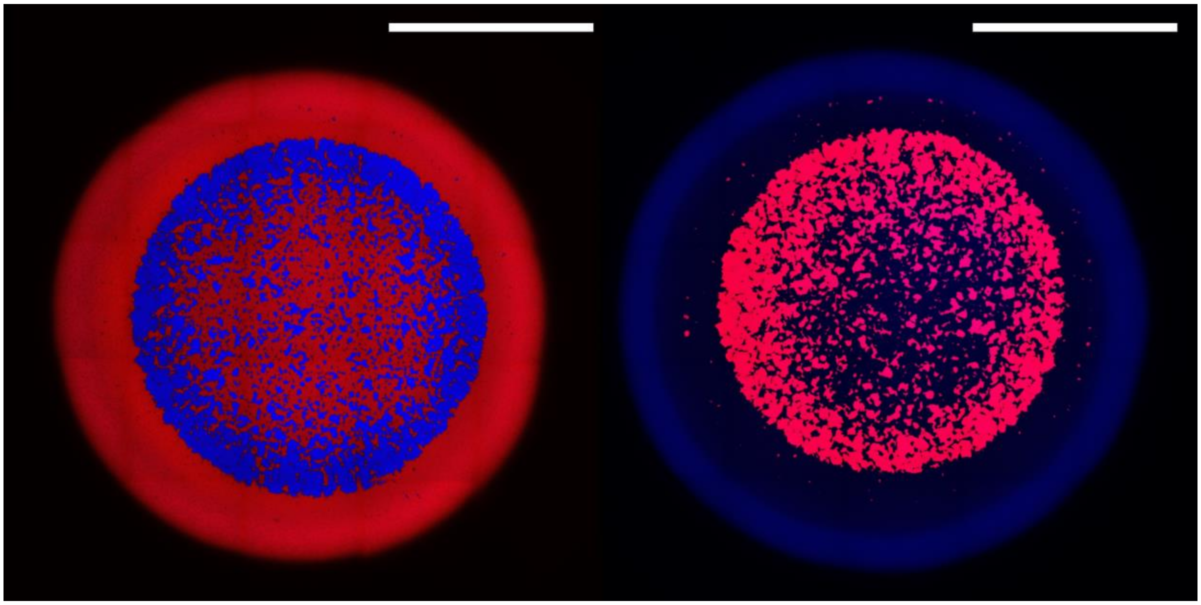

Supplementary Figure 7. Decomposition is not affected by swapping fluorescence markers. On the left we show a phase separated colony of *V. cholerae* strain C6706 in red and 692-79 in blue (as we do throughout the paper, GFP is represented in blue to avoid issues with red-green colourblindness). On the right, we show the same two strains, but with the fluorescent markers reversed. These colonies were grown for 24 h at 25°C. The scale bar denotes 1 mm.

Supplementary Tables

Supplementary Table 1: List of strains used in this study.

Vibrio cholerae strains	Genotype
C6706 red T6SS$^+$	Δ*vc1807*::ptac-*mKO ptac-qstR*
C6706 red T6SS$^-$	Δ*vc1807*::ptac-*mKO ptac-qstR* Δ*vasK*
C6706 green T6SS$^+$	Δ*vc1807*::ptac-*mTFP1 ptac-qstR*
C6706 green T6SS$^-$	Δ*vc1807*::ptac-*mTFP1 ptac-qstR* Δ*vasK*
692-79 red T6SS$^+$	*lacZ*::ptac-*mKO*
692-79 red T6SS$^-$	*lacZ*::ptac-*mKO* Δ*vasK*
692-79 green T6SS$^+$	*lacZ*::ptac-*mTFP1*
692-79 green T6SS$^-$	*lacZ*::ptac-*mTFP1* Δ*vasK*

Supplementary Table 2: Effects of numbers of T6SSs and T6SS effectors on secretome size.

**Fixed Terms**	**Parameter Estimate**	**Lower 95% C.I.**	**Upper 95% C.I.**	P_{MCMC}
Intercept	–4.4661	–4.7097	–4.2588	**<0.0001**
No. T6SSs	0.0322	0.0085	0.0559	**0.0076**
T6SS effectors	0.0105	0.0014	0.0194	**0.0228**
**Random Terms**	**Variance**	**Lower 95% C.I.**	**Upper 95% C.I.**	
Phylogenetic variance	0.0833	0.0611	0.1096	NA
Residual variance	0.0006	0.0002	0.0014	NA

Supplementary Table 3: Effect of number of T6SSs on secretome size.

**Fixed Terms**	**Parameter Estimate**	**Lower 95% C.I.**	**Upper 95% C.I.**	P_{MCMC}
Intercept	–4.4652	–4.717	–4.2313	**<0.0001**
No. T6SSs	0.0456	0.0252	0.0672	**<0.0001**
**Random Terms**	**Variance**	**Lower 95% C.I.**	**Upper 95% C.I.**	
Phylogenetic variance	0.0854	0.0606	0.1102	NA
Residual variance	0.0007	0.0002	0.0014	NA

Supplementary Table 4: Effect of number of T6SS effectors on secretome size.

Fixed Terms	Parameter Estimate	Lower 95% C.I.	Upper 95% C.I.	P_{MCMC}
Intercept	–4.44223	–4.66215	–4.22636	**<0.0001**
T6SS effectors	0.0164	0.0084	0.0242	**<0.0001**
**Random Terms**	**Variance**	**Lower 95% C.I.**	**Upper 95% C.I.**	
Phylogenetic variance	0.085	0.0612	0.1102	NA
Residual variance	0.0007	0.0002	0.0015	NA

Supplementary Table 5: Effects of numbers of T6SSs, T6SS effectors and genome size on secretome size.

Fixed Terms	Parameter Estimate	Lower 95% C.I.	Upper 95% C.I.	P_{MCMC}
Intercept	–4.2362	–5.4144	–3.0159	**<0.0001**
No. T6SSs	0.0326	0.008	0.0564	**0.01**
T6SS effectors	0.0106	0.0021	0.0204	**0.0256**
Log genome size	–0.0279	–0.1777	0.1133	0.7248
**Random Terms**	**Variance**	**Lower 95% C.I.**	**Upper 95% C.I.**	
Phylogenetic variance	0.0838	0.0582	0.1074	NA
Residual variance	0.0007	0.0001	0.0014	NA

Supplementary References

1 Bernardy, E. E., Turnsek, M. A., Wilson, S. K., Tarr, C. L. & Hammer, B. K. Diversity of Clinical and Environmental Isolates of *Vibrio cholerae* in Natural Transformation and Contact-Dependent Bacterial Killing Indicative of Type VI Secretion System Activity. *Applied and Environmental Microbiology* **82**, 2833-2842 (2016).
